# Supplementary material for: Differences and Changes in Cerebellar Functional Connectivity of Parkinson’s Patients with Visual Hallucinations
Source: Brain Sci. 2023 Oct 13;13(10):1458. doi: 10.3390/brainsci13101458 (PMC10605214; doi:10.3390/brainsci13101458)
Supplement: Supplementary file 1 [file brainsci-13-01458-s001.zip › brainsci-2640070-supplementary.pdf]

Supplementary Materials

Table S1. FC statistical analysis results for $p < 0.05$.

<table>
<tr><th colspan="2">PD-H vs. HC</th><th colspan="2">PD-H vs. PD-NH</th></tr>
<tr><td>Node-a</td><td>Node-b</td><td>Node-a</td><td>Node-b</td></tr>
<tr><td>Cerebelum_3_R</td><td>Frontal_Mid_R</td><td rowspan="21">Vermis_1_2</td><td rowspan="21">Precentral_L
Frontal_Mid_L
Frontal_Inf_Oper_L
Frontal_Inf_Oper_R
Frontal_Inf_Tri_L
Frontal_Inf_Tri_R
Cingulum_Mid_R
Postcentral_L
Parietal_Inf_L
Precuneus_L
Precuneus_R</td></tr>
<tr><td>Cerebelum_4_5_L</td><td>Precuneus_L</td></tr>
<tr><td rowspan="2">Cerebelum_4_5_R</td><td>Occipital_Inf_R</td></tr>
<tr><td>Precuneus_L</td></tr>
<tr><td rowspan="2">Cerebelum_6_R</td><td>Postcentral_L</td></tr>
<tr><td>Precuneus_L</td></tr>
<tr><td>Cerebelum_9_L</td><td>Occipital_Inf_R</td></tr>
<tr><td rowspan="7">Cerebelum_9_R</td><td>Frontal_Sup_R</td></tr>
<tr><td>Frontal_Mid_R</td></tr>
<tr><td>Occipital_Inf_R</td></tr>
<tr><td>Postcentral_L</td></tr>
<tr><td>Parietal_Inf_L</td></tr>
<tr><td>Precuneus_L</td></tr>
<tr><td>Temporal_Sup_L</td></tr>
<tr><td>Vermis_3</td><td>Frontal_Mid_R</td></tr>
<tr><td>Vermis_1_2</td><td>Occipital_Inf_R</td></tr>
<tr><td rowspan="4">Vermis_4_5</td><td>Occipital_Inf_R</td></tr>
<tr><td>Postcentral_L</td></tr>
<tr><td>Parietal_Inf_L</td></tr>
<tr><td>Precuneus_L</td></tr>
<tr><td>Vermis_8</td><td>Occipital_Inf_R</td></tr>
<tr><td colspan="2">PD-NH vs HC</td><td></td><td></td></tr>
<tr><td>Cerebelum_9_R</td><td>Temporal_Sup_L</td><td></td><td></td></tr>
<tr><td rowspan="8">Vermis_1_2</td><td>Precentral_L</td><td></td><td></td></tr>
<tr><td>Frontal_Inf_Oper_L</td><td></td><td></td></tr>
<tr><td>Frontal_Inf_Oper_R</td><td></td><td></td></tr>
<tr><td>Frontal_Inf_Tri_L</td><td></td><td></td></tr>
<tr><td>ParaHippocampal_L</td><td></td><td></td></tr>
<tr><td>Angular_R</td><td></td><td></td></tr>
<tr><td>Cerebelum_4_5_L</td><td></td><td></td></tr>
<tr><td>Cerebelum_4_5_R</td><td></td><td></td></tr>
</table>
